# Supplementary material for: Differentiation Capacity of Bone Marrow-Derived Rat Mesenchymal Stem Cells from DsRed and Cre Transgenic Cre/loxP Models
Source: Cells. 2022 Sep 5;11(17):2769. doi: 10.3390/cells11172769 (PMC9455627; doi:10.3390/cells11172769)
Supplement: Supplementary file 1 [file cells-11-02769-s001.zip › cells-1859375-supplementary.pdf]

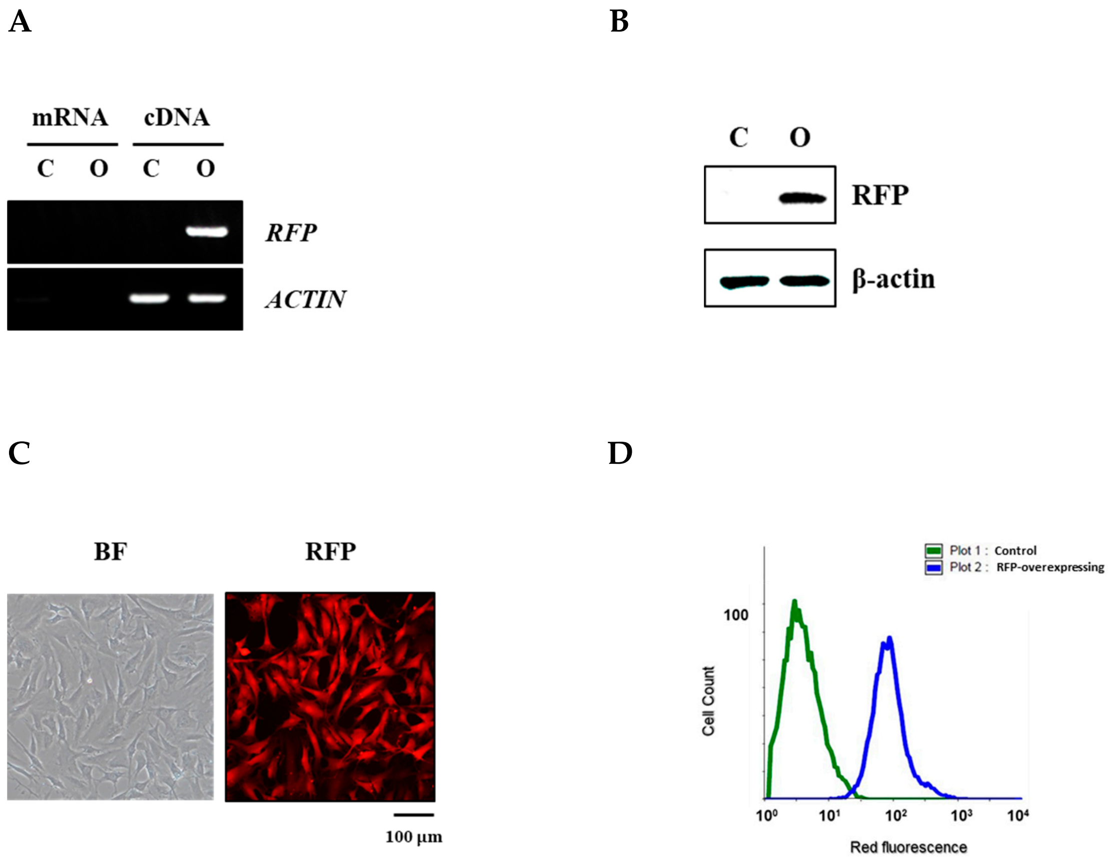

Figure S1. RFP overexpression in rat BM-MSCs. (A) RT-PCR reveals the mRNA expression level of control (WT) and RFP-overexpressing MSCs. (B)Western blotting indicating RFP (27 kDa) and internal control beta-actin (34 kDa) in control and RFP overexpressing cells. (C) Fluorescence microscopy reveals the signal for RFP (red). (D) The cells were examined by using flow cytometry at the 588 nm wavelength to determine the intensity of RFP. Scale bars, 100 µm. C, control; O, RFP-overexpressing group.

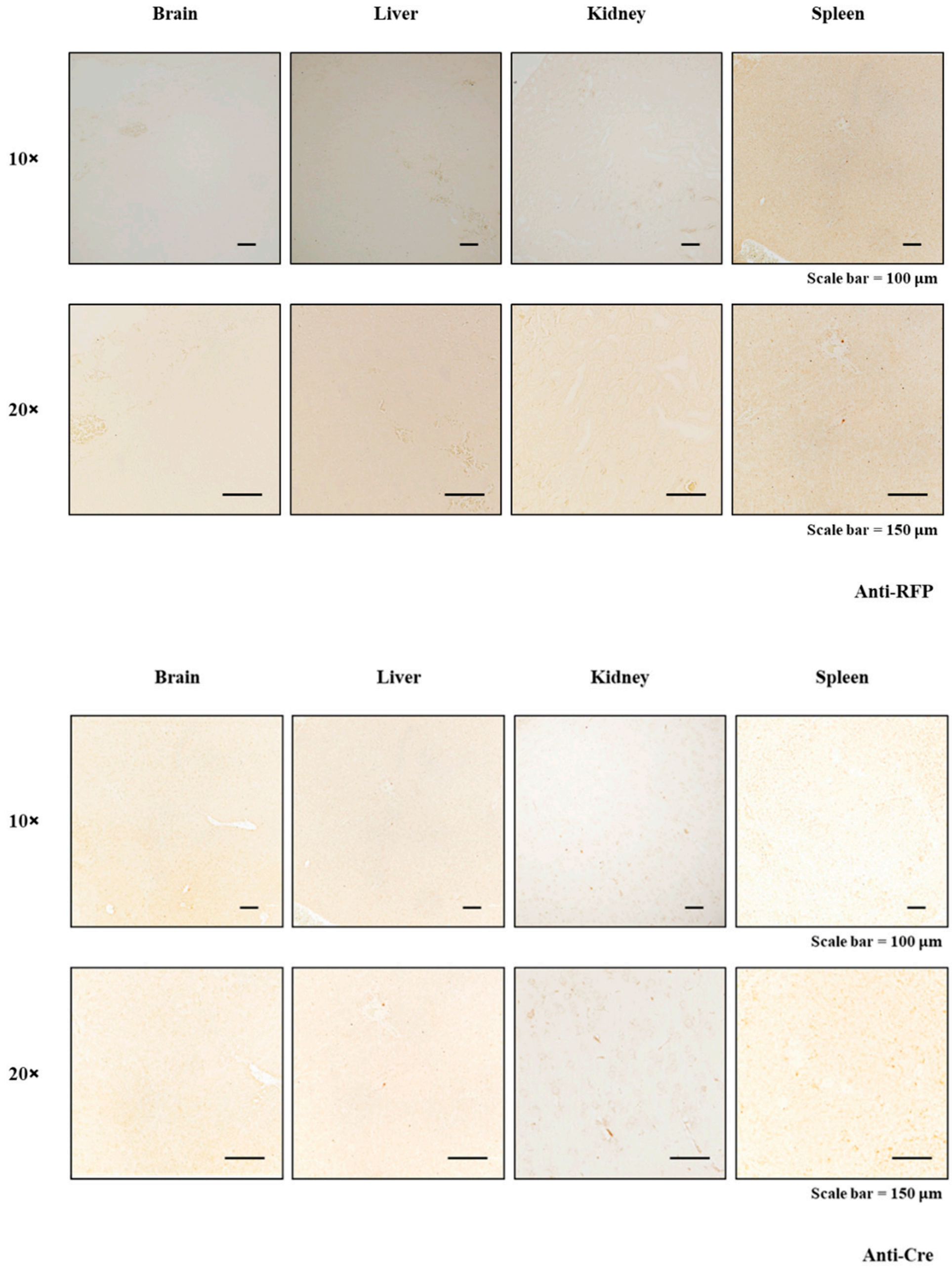

Figure S2. Histological analysis of tissue organs in wild-type (WT) rat. Chromogenic results of distinct organ sections that were collected from WT rats. Specimens stained with RFP and Cre antibodies respectively served as a negative control. Scale bars,100 µm (10×), 150 µm (20×).

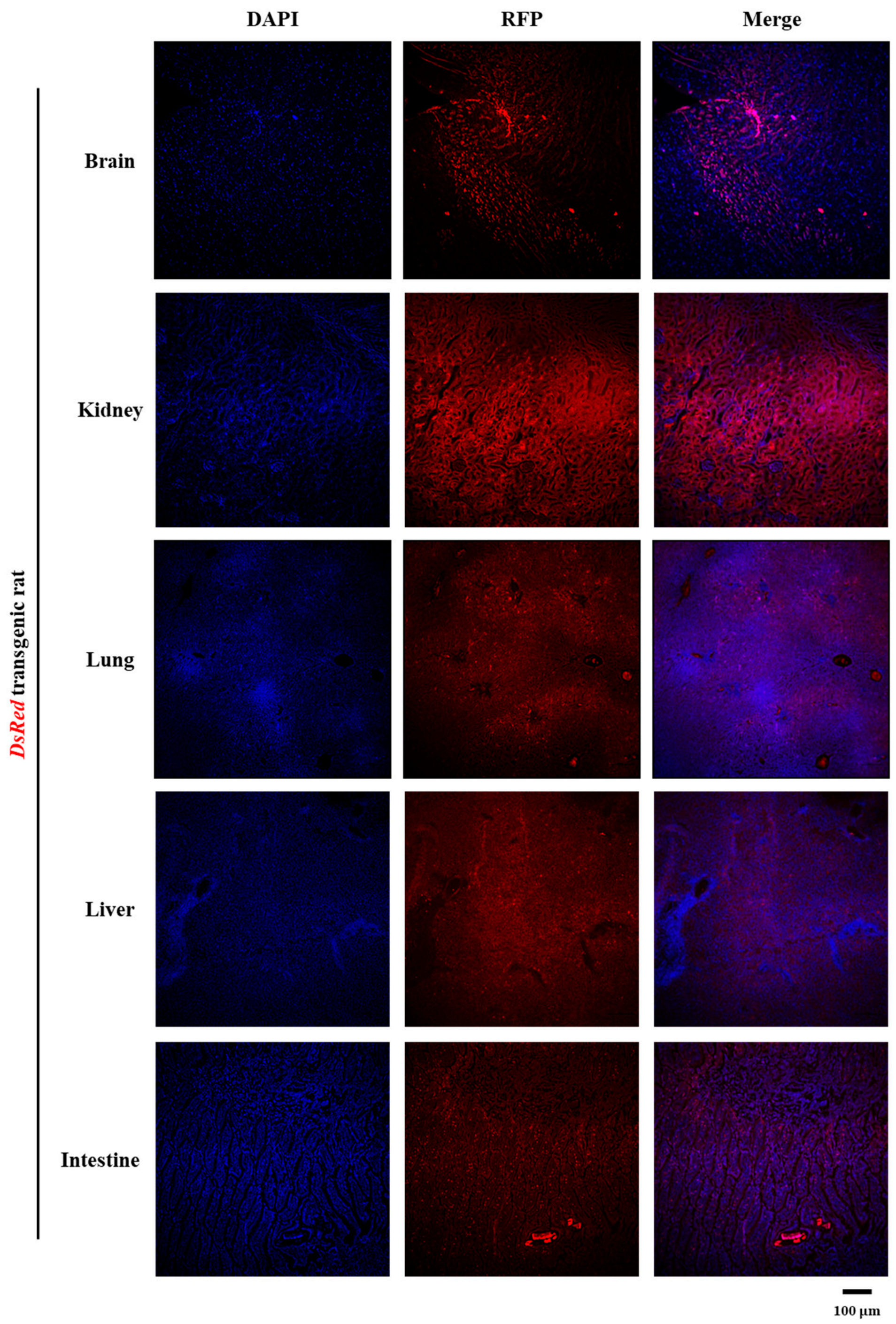

Figure S3. IHC analysis of tissue organs in DsRed transgenic rats through visualization of fluorescent detection. Red fluorescence of these organ sections was observed by the Nikon G-2A filter set. The nucleus was stained with DAPI. Scale bars, 100 μm.

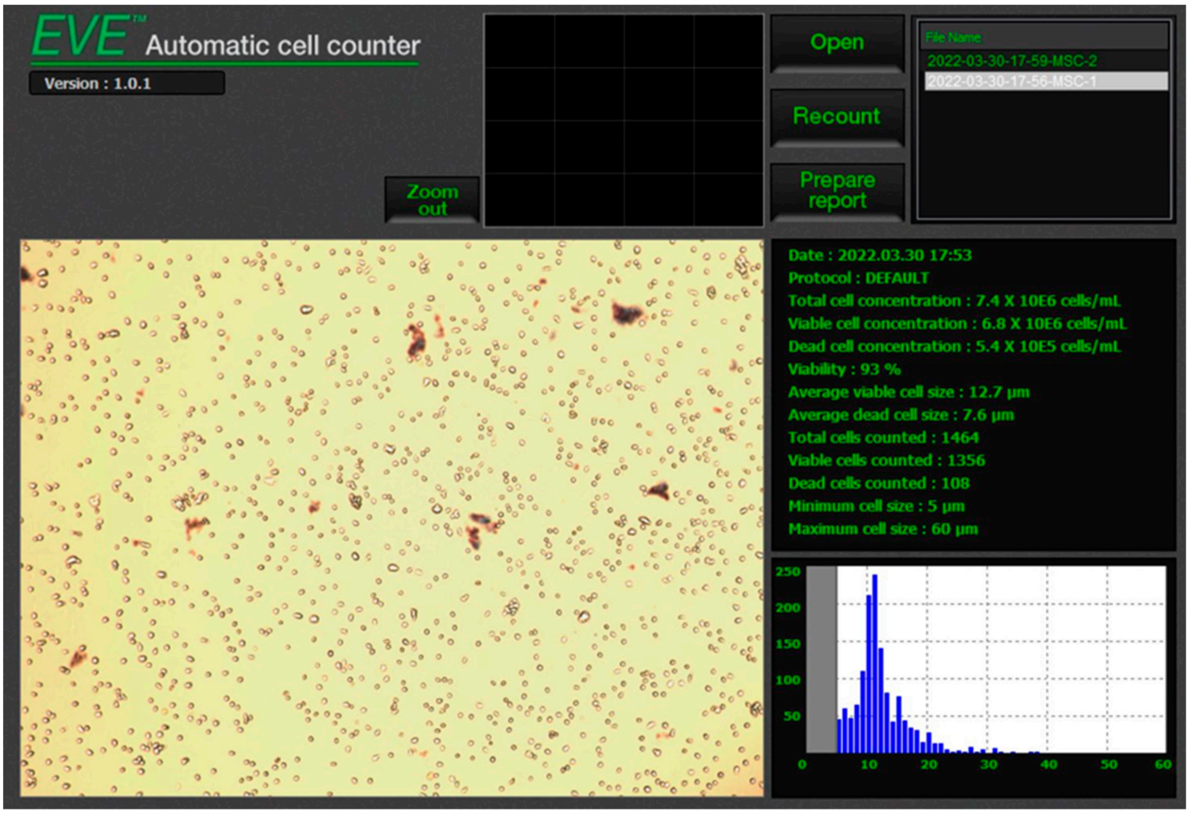

Figure S4. Schematic diagram of the analytical data for cell counting. The *EVE™* automatic cell counter uses state-of-the-art optics and image analysis for automatic cell counting. It was designed to measure cell count and viability (live, dead, and total cells) accurately and precisely using the standard trypan blue technique. All data presented in this article were acquired by this instrument.

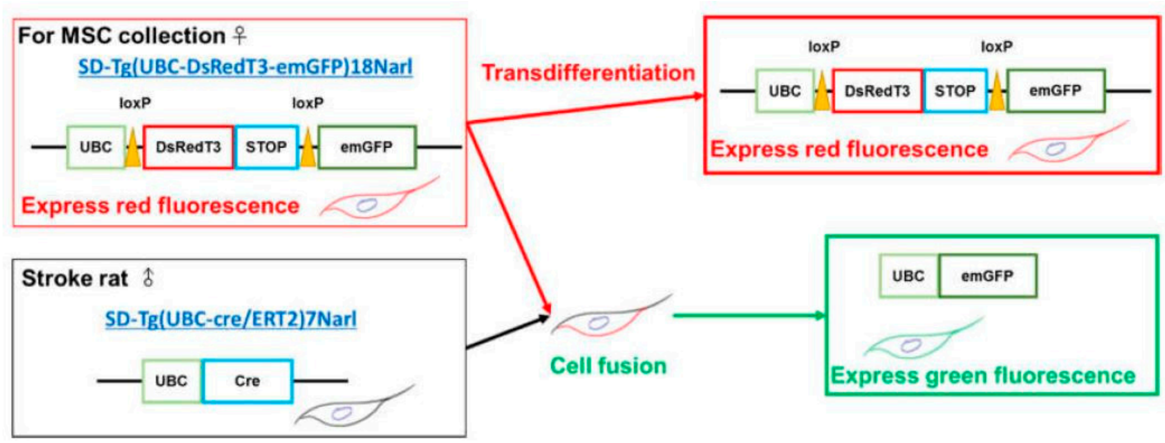

Figure S5. Cre and *loxP* system for testing the hypothesis of cell fusion or transdifferentiation in transgenic models. If cell fusion occurs, the MSCs which we injected will turn to express GFP instead of RFP. In that case, the cell fusion could be validated by detecting the expression of GFP.

Weeks	DsRed-Male			DsRed-Female		
X	C:Y1	C:Y2	C:Y3	D:Y1	D:Y2	D:Y3
3	51.4	54.0	56.6	53.2	52.1	50.8
4	90.8	92.0	94.6	75.4	78.3	72.6
5	130.0	126.0	123.0	92.4	97.3	100.2
6	149.0	167.0	154.0	125.0	128.0	132.0
7	199.0	200.0	204.0	139.0	143.0	147.0
8	213.0	210.0	215.0	163.0	165.0	169.0
9	229.0	237.0	224.0	172.0	175.0	179.0
10	232.0	228.0	240.0	181.0	182.0	180.0
11	233.0	238.0	243.0	181.3	182.2	183.4
12	242.0	251.0	246.0	184.2	187.0	185.0

Weeks	Cre-Male			Cre-Female		
X	E:Y1	E:Y2	E:Y3	F:Y1	F:Y2	F:Y3
3	50.3	53.4	52.8	48.3	49.4	51.5
4	89.3	90.2	88.7	74.3	72.5	69.7
5	122.0	130.0	124.0	87.7	88.6	89.1
6	142.0	153.0	148.0	106.3	107.4	110.2
7	178.0	185.0	182.0	128.5	124.6	132.1
8	195.0	201.0	193.0	148.0	154.0	143.0
9	202.0	213.0	208.0	152.0	163.0	157.0
10	211.0	219.0	214.0	165.0	172.0	169.0
11	218.0	227.0	221.0	170.0	174.0	177.0
12	225.0	231.0	228.0	176.0	180.0	181.0

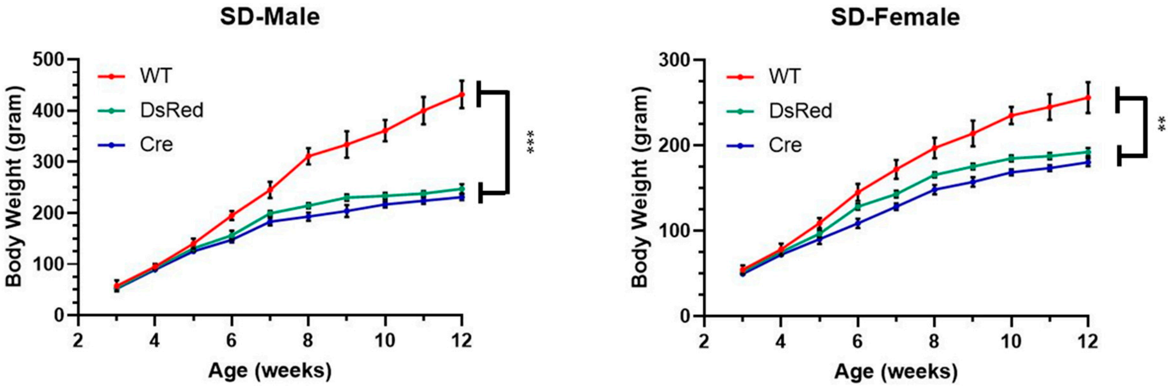

Table S1. Weight to age-in-week comparison for wild-type and transgenic rats. Statistical analyses were carried out using analysis of variance (ANOVA) with GraphPad Prism software. The confidence interval was 95% considering the multiple comparisons between groups. ** $p < 0.01$, *** $p < 0.001$.

Accession no.	Name	Sequence	Product size (bp)
NP_445817	aP2	F: 5′-GCGTGGAATTCGATGAAATCA-3′<br>R: 5′-CCCGCCATCTAGGGTTATGA-3′	68
NP_001099820	DKK1	F: 5′-TCCGTCTGCCTCCGATCATC-3′<br>R: 5′-GCCTTTCCGTTTGTGCTTGG-3′	123
NP_037061	Collagen type II	F: 5′-CCAGGTCCTGCTGGAAAA-3′<br>R: 5′-CCTCTTTCTCCGGCCTTT-3′	68
XP_038945735	β-actin	F: 5′-CGCCAACCGCGAGAAGAT-3′<br>R: 5′-CGTCACCGGAGTCCATCA-3′	134
QFU20097	DsRed	F: 5′-CAGTACGGCTCCAAGGTGTA-3′<br>R: 5′-GTGTAGTCCTCGTTGTGGGA-3′	400
YP_006472	Cre	F: 5′-CTAAACATGCTTCATCGTCGGTC-3′<br>R: 5′-TCTGACCAGAGTCATCCTTAGCG-3′	500
QPL12221.1	RFP	F: 5′-TGAGAATCAAGGTGGTCGAG-3′<br>R: 5′-CGTCAGCGGGGTACAGCATC-3′	333

Table S2. Primers used for reverse transcription polymerase chain reaction for specific gene expression.
